# Influence of *Staphylococcus aureus* Infection on Partially Ischemic Excisional Skin Wounds

**DOI:** 10.1155/2024/2281747

**Published:** 2024-09-21

**Authors:** Adrian E. Rodrigues, David M. Dolivo, Chun Hou, Yingxing Li, Lauren S. Sun, Thomas A. Mustoe, Seok Jong Hong, Robert D. Galiano

**Affiliations:** ^1^ Department of Surgery, Division of Plastic Surgery Northwestern University Feinberg School of Medicine, Chicago, USA; ^2^ Department of Plastic and Cosmetic Surgery First Affiliated Hospital of Guangzhou Medical University, Guangzhou, China

## Abstract

**Background:**

Skin wounds, whether medically or incidentally induced, are always at a risk of becoming infected, but the infection risks are greater when the wounds are recovering under ischemic, poorly perfused conditions. *Staphylococcus aureus,* which frequently infects cutaneous and soft tissue, can infect to a greater extent when wounds are poorly perfused. Bad as this may be, both MSSA and MRSA strains of *S. aureus* can cause severe infections, with MRSA being considered more aggressive.

**Methods:**

In this study, we used a lagomorph ear excisional wound model to initially test the influence of partial ischemia on uninfected wound healing. We then subsequently test the same ischemic injury model under an active MSSA infection and compared these wounds against normally perfused MSSA-infected wounds. Lastly, we test whether differences in healing exist between MSSA-infected and MRSA-infected wounds, both under the same ischemic model.

**Results:**

The data suggest that partial ischemia considerably reduces healing of noninfected wounds (epithelial gap *P*=^∗∗∗∗^, granulation gap *P*=^∗∗∗^, and granulation area *P*=^∗∗∗∗^). Similarly, partial ischemic wounds coupled with MSSA infection display healing impairments against likewise-infected wounds healing under normal perfusion (epithelial gap *P*=^∗^, granulation gap *P*=^∗^, and granulation area *P*=^∗∗^). No significant differences were observed between MSSA-infected and MRSA-infected wounds healing under ischemia.

**Conclusion:**

The data produced quantitative differences in healing under various conditions consequent to ischemia and *S. aureus* infection. Although it is well recognized that ischemia and infection adversely influence healing, by testing these conditions, we determined the detrimental magnitude such circumstances inflict on skin healing, thereby providing a relative reference to compare and gauge when met with similar conditions clinically.

## 1. Introduction

Surgical site infections are common nosocomial occurrences, and healthcare personnel have been attempting to prevent them for years [1, 2]. In spite of this, multidrug-resistant bacteria have also been steadily growing [3], and continued emergence of resistance may perpetuate this problem indefinitely. This climb in infections has also caused additional burdens for those at the forefront of healthcare. Aside from managing all surgeries with strict aseptic techniques and discretionally utilizing prophylactic antibiotics, healthcare personnel are also expected to further assess the risk to those most vulnerable to infection [4]. Yet even in the absence of infection, ischemic wounds show deficiencies in healing and present difficulties in management. Due to decreases in blood delivery, wounds that are poorly perfused can course with problems in hemostasis, angiogenesis, proliferation, re-epithelialization, and/or remodeling—processes essential for wound regeneration [5–8]. Outside of surgically induced wounds, diabetic foot ulcers reputably show disturbances with one or more of these stages, commonly growing wider over time and regularly becoming infected [9]. In experimental models involving stroke and chronic wounds, microcirculatory and local inflammatory challenges have been revealed, exposing disruptions in endothelial function that cascade into local immunodepression and infection risk [10, 11].

Therefore, in animal research, perfusion-restricted wound models come closest to mirroring such conditions, and such models can be derived by dividing either the vascular and/or arterial vessels supplying and draining a targeted area [12, 13]. In this study, we used a lagomorph (rabbit) excisional ear-skin wound model and impose the wounds to heal under partially ischemic conditions by dividing the ears' central artery at its base. This was the hallmark of the wound model, and it was repeatedly used in three substudies incorporating noninfection and infection conditions. Specifically, we utilized two *Staphylococcus aureus* strains to induce infections—UAMS-1, which is methicillin-sensitive (MSSA) [14], and USA300, which is methicillin-resistant (MRSA) [15]—and analyzed the influence UAMS-1 infections cause on partially ischemic compared to nonischemic wounds, and further tested unilateral yet coinciding infections of both UAMS-1 and USA300 under the same ischemic model and compared their impact on wound closure.

This investigation was inspired by gaps in research that have tested ischemia paired with these microorganisms under an animal model that similarly aligns with human skin healing, and it was conducted with a hypothesis-free methodology to establish discovery and contextual understanding.

## 2. Materials and Methods

### 2.1. Animal Information, Wounding, and Bacterial Utilization

This project was approved by the Northwestern University Institutional Animal Care and Use Committee. Female New Zealand White rabbits (Envigo, Indianapolis, IN) aged between 17 and 22 weeks were utilized for this study. Anesthetic planes were achieved with intramuscular injections of 40 mg/kg of ketamine and 5 mg/kg of xylazine, with analgesic control succeeding with 0.2 mg/kg of buprenorphine SR along with local subcutaneous 1% lidocaine-epinephrine to the wounds of each ear. Anesthetic plane reversal was achieved with 0.5 mg/kg of atipamezole.

**Figure 1:**
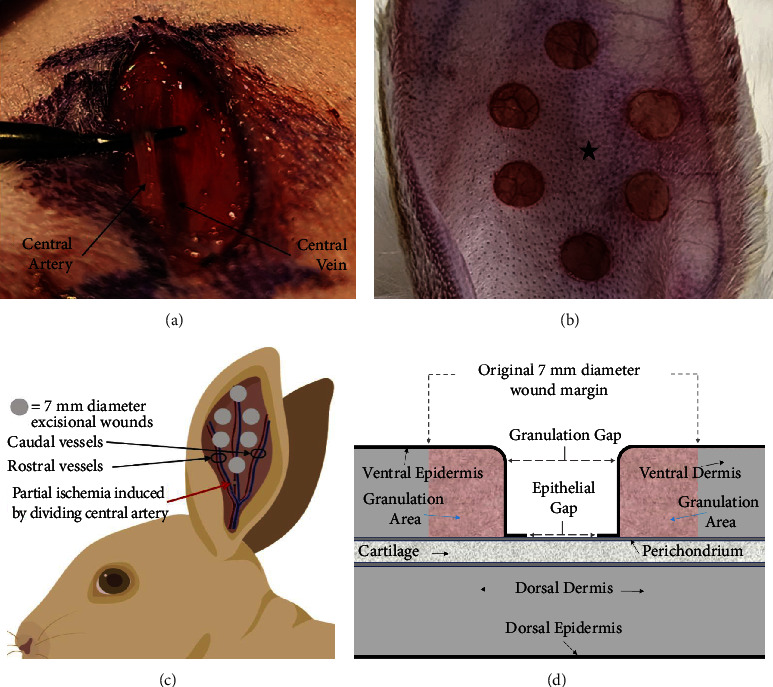
Animal wound model and histological healing parameters: (a) ischemia induction procedure, showing isolation of the ears' dorsal artery from its vein prior to its division. (b) Six skin wounds on ventral surface of ear on day zero, measuring 7 mm in diameter (star denotes area utilized for oxygen measurements, see Figure 2). (c) Illustration depicting the entire ear, its three main vessel branches, the location of the skin wounds, and the central artery's location of division relative to the six skin wounds. (d) Illustration of a harvested cross-sectional skin wound as viewed histologically, showing partial healing. Parameters used to quantify healing are the epithelial gap (epidermis), granulation gap (open wound space), and the granulation area (portion of wound that has undergone healing and therefore contains granulation tissue).

Partial ischemia (henceforth referred to as ischemia) was induced by incising the dorsal skin at the base of the ear, dissecting the central artery free, dividing it with electrosurgery, and reapproximating the overlying skin with 5-0 prolene (Ethicon, Cincinnati, OH). Sham control involved only incising the dorsal skin directly above the basal central vessels, leaving all blood vessels uninjured, and reapproximating the skin with prolene. All animals underwent bilateral ear excisional skin wounds with a 7-mm diameter biopsy punch (Acuderm, Ft. Lauderdale, FL), whereby the ventral skin was excised down to the perichondrium. In a single subset experiment, skin partial pressure of oxygen (pO_2_) was measured with an Oxylite 2000 fiber-optic sensor (Oxford Optronix, Adderbury, United Kingdom) placed central to the 6 skin wounds (Figures 1(a), 1(b) and 1(c)).

In substudies incorporating infection, wounds were inoculated with either UAMS-1, a MSSA clinical isolate [14], or USA300 JE2, a MRSA clinical isolate [15]. For antibiotic resistance testing, overnight bacterial cultures were used to inoculate fresh tubes of LB (Luria-Bertani) broth containing various concentrations of oxacillin (Sigma-Aldrich, St. Louis, MO), which were then incubated in bacterial shakers at 37°C. Growth curves were established by collecting samples at defined time points, placing them on ice, and using a SmartSpec Plus spectrophotometer (Bio-Rad, Hercules, CA) to obtain OD600 values relative to blank solution readings of sterile LB (Figure 3). Inoculation involved pipetting 10 *µ*l (equivalent of 10^7^ colony forming units (CFUs)) of bacterial culture onto each wound, followed with an application of Tegaderm (3M, Maplewood, MN) film to each ear to support infection and prevent desiccation.

### 2.2. Tissue Harvest, Histology, and Quantification of Wound Healing Parameters

For histology, individual wound samples were extracted with a 10-mm biopsy punch and fixed in 10% neutral-buffered formalin. Tissues were then serially dehydrated, embedded in paraffin, and cut to 5 *μ*m-thick sections. Sectioned samples were then deparaffinized and serially hydrated and stained with hematoxylin and eosin (H&E) according to standard protocols, and histological healing parameters and images were obtained using a Nikon Eclipse 50i microscope linked to a computer running Nikon NIS Elements software (Nikon Instruments, Melville, NY). Calculated wound healing parameters included epithelial gap, granulation gap, and granulation area and were measured as depicted in Figure 1(d). Impaired wound healing was characterized by increased epithelial and granulation gaps, along with a reduced granulation area. Conversely, enhanced wound healing exhibited the opposite findings. These metrics provide clear criteria for assessing and comparing wound healing progress.

### 2.3. Induced Partial Ischemic Wounds vs. Sham Control Wounds: Primary Substudy

To determine if skin wounds healing under induced ischemia develop healing delays, we assessed the healing of ischemic wounds and compared them against skin wounds healing under normally perfused conditions (sham control), both under noninfected environments. On postoperative day (POD) 0, all animals underwent bilateral excisional skin wounding, 6 per ear, with one ear undergoing central artery division (induced ischemia), and the contralateral ear allocated to sham control. Skin wounds were allowed to progress infection-free until POD 7, at which time wounds were harvested for analysis. Given that noninfected sham-control wounds traditionally heal rapidly, histological assessment was only possible prior to wound closure, and therefore, tissue harvest necessitated a day 7 time point.

### 2.4. Induced Partial Ischemic Wounds Infected with UAMS-1 vs. Sham Control Wounds Infected with UAMS-1: Secondary Substudy

In order to test whether UAMS-1-infected ischemic wounds demonstrated greater reductions in healing compared to normally perfused infected wounds, we tested both conditions in the same animal model. On POD 0, all animals underwent bilateral excisional skin wounds, 6 per ear, with one ear undergoing central artery division (induced ischemia), and the contralateral allocated to sham control (normal perfusion). On POD 3, all wounds were inoculated with UAMS-1, and infections were allowed to progress until POD 10, at which point, tissues were harvested.

Due to the rinsing away of bacteria from profuse bleeding and transudate, inoculation was postponed until day 3 to achieve successful infection and biofilm assembly within the wound bed. Furthermore, given that infected wounds exhibited delayed healing, analyzing on day 10 was well suited. This allowed the infection to progress for several days prior to complete healing, facilitating histological wound assessments.

### 2.5. Induced Partial Ischemic Wounds Infected with UAMS-1 vs. Induced Partial Ischemic Wounds Infected with USA300: Tertiary Substudy

To compare ischemic healing between wounds infected with MSSA (UAMS-1) and MRSA (USA300) strains, we tested both conditions using the same animal model. On POD 0, all animals underwent bilateral excisional skin wounds, 6 per ear, and bilateral central artery division (induced ischemia). On POD 3, each animal received inoculations of UAMS-1 to all skin wounds on one ear, while wounds on the contralateral ear were inoculated with USA300. Wounds were allowed to progress until POD 10, at which time, tissues were harvested.

### 2.6. Skin Oxygenation Measurements

In the tertiary substudy mentioned directly above, we measured skin oxygenation in order to quantify ischemic severity and recovery. Under the study, all animals underwent bilateral measurements of partial pressure of oxygen (pO_2_) on five occasions throughout the study, with measurements taken central to the wounds (Figure 1(b)).

### 2.7. Statistical Analyses

Statistical analyses were performed using Prism 9 (GraphPad, San Diego, CA). All summary statistics are represented as the mean ± standard deviation, *n* = number of independent skin wounds, ^∗^*P* < 0.05, ^∗∗^*P* < 0.01, ^∗∗∗^*P* < 0.001, ^∗∗∗∗^*P* < 0.0001, and not significant (ns) *P* > 0.05. Skin oxygenation statistical comparisons were performed with an ordinary one-way ANOVA with Dunnett's post-hoc pairwise comparisons, comparing groups to “Day 0 baseline” (pO_2_ measured immediately prior to vessel division). Excisional wound statistical comparisons were made using two-tailed, unpaired Student's *t*-tests.

## 3. Results

### 3.1. Induced Partial Ischemic Wounds vs. Sham Control Wounds: Primary Substudy

In this primary subexperiment, discernible wound differences could be grossly observed between the sham control and induced ischemia group on the day of harvest, revealing that noninfected ischemic wounds had stunted healing compared to uninfected, normally perfused sham control wounds (Figures 4(a) and 4(b)). Similarly, histological measurements quantifying epithelial gap (*P*=^∗∗∗∗^), granulation gap (*P*=^∗∗∗^), and granulation area (*P*=^∗∗∗∗^) revealed significant differences between the groups, demonstrating that wound healing was significantly slowed in response to partial ischemia (Figures 4(c), 4(d), and 4(e)).

### 3.2. Induced Partial Ischemic Wounds Infected with UAMS-1 vs. Sham Control Wounds Infected with UAMS-1: Secondary Substudy

In this secondary subexperiment, gross observational differences in healing were also discernible between the tested groups, showing that ischemic UAMS-1 infected wounds experience greater healing delays when compared to normally perfused UAMS-1 infected wounds (Figures 5(a) and 5(b)). Gross observations were further substantiated by histological data, whereby quantification of healing parameters demonstrated statistically significant differences in epithelial gap (*P*=^∗^), granulation gap (*P*=^∗^), and granulation area (*P*=^∗∗^) (Figures 5(c), 5(d), and 5(e)).

### 3.3. Induced Partial Ischemic Wounds Infected with UAMS-1 vs. Induced Partial Ischemic Wounds Infected with USA300: Tertiary Substudy

This tertiary subexperiment did not demonstrate any gross observational differences between ischemic wounds infected with UAMS-1 and USA300. There were also no significant differences in epithelial gap, granulation gap, or granulation area between ischemic UAMS-1-infected wounds and ischemic USA300-infected wounds (Figures 6(a), 6(b), 6(c), 6(d), and 6(e)).

### 3.4. Skin Oxygenation Measurements

**Figure 2:**
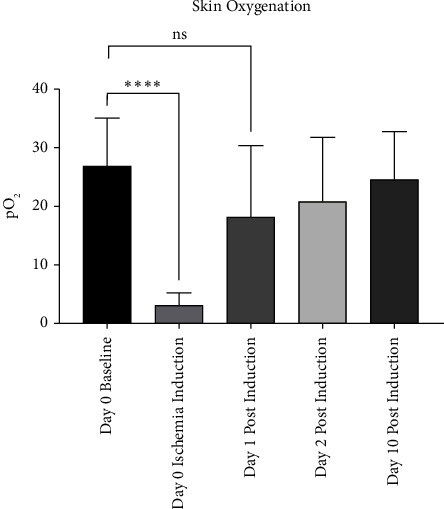
Skin oxygenation measurements. Skin oxygen partial pressure graphical data, showing day 0 baseline (levels immediately prior to dividing the central artery) and day 0 ischemia induction (immediately after dividing the central artery), of which a significant drop was determined between these two measurements. Partial pressures on days 1, 2, and 10 postinduction show the return of oxidation over time, with all three of these postinduction measurements showing no statistical significance when compared to day 0 baseline. This suggests that soon after the arteries were divided to induce ischemia, oxygenation recovered within 24 hours. This figure was generated from one independent experiment described as the tertiary substudy. Skin oxygenation partial pressure was chosen due to its practicality, including its ability to immediately and progressively capture distinctions secondary to perfusion attenuation.

**Figure 3:**
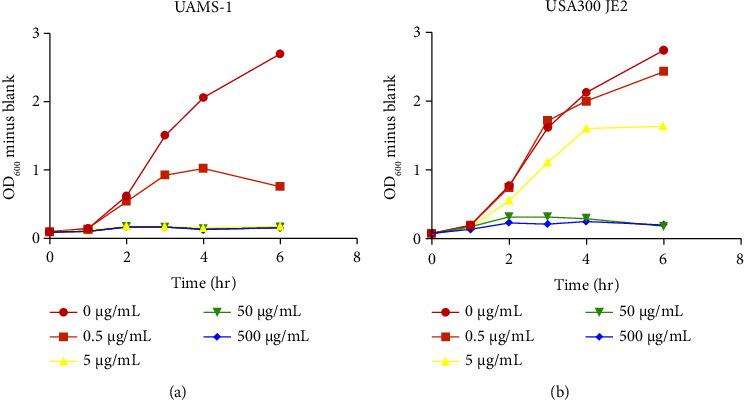
*S. aureus* growth curves under oxacillin concentrations: (a) methicillin-sensitive *S. aureus* strain UAMS-1, showing growth reduction at 0.5 *μ*g/ml of oxacillin and growth inhibition at 5, 50, or 500 *μ*g/ml. (b) Methicillin-resistant *S. aureus* strain USA300 JE2, showing continued growth at 0.5 *μ*g/ml of oxacillin, minor reduction at 5 *μ*g/ml, and growth inhibition at 50 or 500 *μ*g/ml. Each figure was generated from one independent experiment, with time frame and concentrations chosen to produce the most discernible differences.

**Figure 4:**
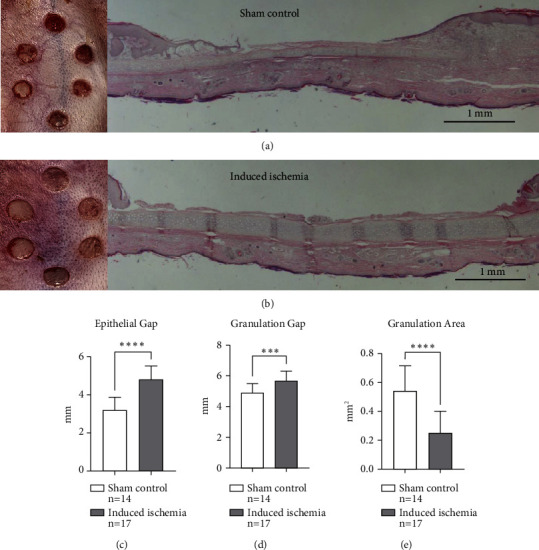
Induced partial ischemic wounds vs. sham control wounds: primary substudy. (a) Gross wound healing under normally perfused conditions (sham control) on the day of harvest, along with an accompanying histological image showing ventral skin (top) healing. (b) Gross wound healing under ischemic conditions (induced ischemia) on the day of harvest, along with an accompanying histological image showing ventral skin wound with reduced healing. Wound healing under ischemic conditions show a significantly greater epithelial gap (c), greater granulation gap (d), and a reduced granulation area (e), all of which imply reduced healing. This figure was generated from one independent experiment described as the primary substudy. The choice of the wounding parameters (epithelial gap, granulation gap, and granulation area) was chosen since each conveys distinctive healing parameters whereby when taken together, judgement on a wound's progress can be made.

**Figure 5:**
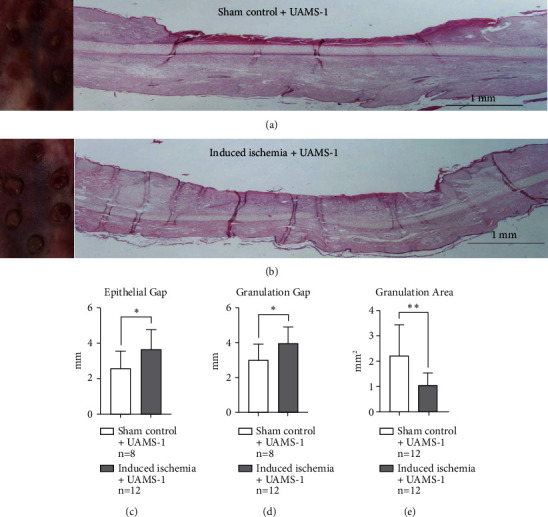
Induced partial ischemic wounds infected with UAMS-1 vs. sham control wounds infected with UAMS-1: secondary substudy. (a) Gross wounds under normally perfused conditions coupled with sensitive *S. aureus* infection (sham control + UAMS-1) on the day of harvest, along with an accompanying histological image. (b) Gross wound healing under ischemic conditions coupled with sensitive *S. aureus* (induced ischemia + UAMS-1) on the day of harvest, along with an accompanying histological image. Grossly, ischemic wounds (b) show reduced healing. Wound healing under ischemic conditions show a significantly greater epithelial gap (c), greater granulation gap (d), and a reduced granulation area (e), all of which imply reduced healing. This figure was generated from one independent experiment described as the secondary substudy. The choice of the wounding parameters was chosen since each conveys distinctive healing parameters whereby when taken together, judgement on a wound's progress can be made.

**Figure 6:**
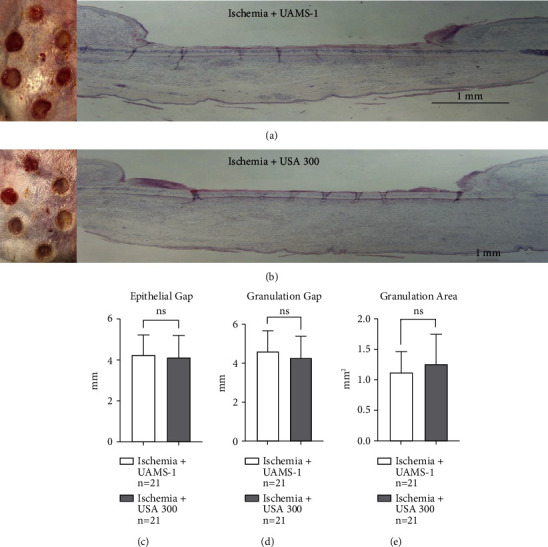
Induced partial ischemic wounds infected with UAMS-1 vs. induced partial ischemic wounds infected with USA300: tertiary substudy. (a) Gross wounds under ischemic conditions coupled with sensitive *S. aureus* infection (ischemia + UAMS-1) on the day of harvest, along with an accompanying histological image. (b) Gross wound healing under ischemic conditions coupled with resistant *S. aureus* (ischemia + USA300) on the day of harvest, along with an accompanying histological image. Notice both tested conditions show no discernible healing differences. Also, calculated histological healing parameters, epithelial gap (c), granulation gap (d), and granulation area (e) unsuccessfully revealed statistical differences. This figure was generated from one independent experiment described as the tertiary substudy. The choice of the wounding parameters was chosen since each conveys distinctive healing parameters whereby when taken together, judgement on a wound's progress can be made.

Compiled skin pO_2_ averages taken from ears under the tertiary subexperiment did support that the ischemia created by dividing the central artery had a direct influence on tissue oxygenation. Differences in pO_2_ between Day 0 baseline readings and immediately after central artery division (Day 0 ischemic induction) demonstrated a highly significant reduction (*P*=^∗∗∗∗^). However, there were no statistical differences between Day 0 baseline and subsequent Day 1, Day 2, and Day 10 postinduction readings (Figure 2).

## 4. Discussion

Both the degrees and causes of ischemia vary considerably. Some, like Raynaud's phenomenon, are mild and only cause undertone changes during stress or temperature fluctuations [16]. But others like diabetic peripheral artery disease are more severe and can lead to ulcer formation when glucose is poorly controlled. Nevertheless, outside of the central nervous system, medical conditions that subtly alter perfusion generally have little to no effect. This is because the skin and other organ systems have abundant collateral and choke vessels, allowing blood to be shunted and rerouted efficiently and rapidly [17]. If a condition does linger and persistently reduces perfusion, however, regions local to the ailment may undergo vasodilation and/or angiogenesis to increase regional blood supply. However proficient these physiological solutions are, the effects of ischemia on excisional wound healing in the skin appears to have a lasting impact, as seen in our primary substudy comparing healing of noninfected ischemic and sham skin wounds. In this experiment, dividing one of the three main arterial conduits had a sizable influence on all healing metrics we quantified. The results demonstrate the worth of a stable and consistent blood supply—as seen by the sham control wounds that showed abundant recovery—and the cost of hindering that supply—as noted in the wounds forced to heal under partially ischemic conditions. Although the ischemic ears likely undertook physiological means to counter the perfusion ailments (as observed by the tissue oxygen measurements conducted in the tertiary substudy, Figure 2), the stress induced by the wound healing demands was, at least initially, met short due to a deficient supply, setting the stage for delayed wound healing results.

Commonly, skin wounds that prolong wound healing secondary to an underlying ischemic skin disease run a higher risk of infectivity, and *S*. *aureus* is frequently catalogued in this regard. Although some of its ailments include sepsis, endocarditis, pneumonia, and osteomyelitis, *S. aureus* may also infect the skin locally, resulting in abscess formation or driving skin wound chronicity through establishment of infection [18]. Therefore, we also sought to test whether ischemia boosted the virulence of a methicillin-sensitive strain of *S. aureus*. Upon capturing the data, differences between normally perfused infected wounds (sham control + UAMS-1) and ischemic infected wounds (induced ischemia + UAMS-1) were statistically significant, demonstrating that ischemia further delays healing under conditions of *S. aureus* infection. This result also supports other findings showing that diabetic peripheral arterial disease, when coupled with infection, is likely to worsen and therefore undergo amputation more frequently [19].

In poorly perfused conditions, skin infections are undoubtedly problematic but, under these same conditions, it is not fully known whether infection with a methicillin-resistant strain of *S. aureus* is more detrimental to healing than infection with a methicillin-sensitive strain. Outside of skin infections, there is much literature proposing differences between MRSA and MSSA pathogens. Patients with MRSA osteomyelitis are more likely to undergo surgical procedures, have increased body temperature, and present with higher white blood cell counts [20]. It has also been found that MRSA produces more enterotoxin A and coagulase and demonstrates decreased adherence to fibrinogen, while MSSA produces more enterotoxin C, toxic shock syndrome toxin 1, and adheres better to type I collagen [21]. Although differences between the two types of pathogen have been recorded, some studies describe minimal differences in their virulence [20–23]. Within the United States, MRSA is one of the most common causes of soft tissue and skin infections [24], and lagomorph studies comparing MRSA and MSSA under ischemic excisional wound conditions have not been published to date, to the best of our knowledge. Therefore, we decided to perform these studies to address this gap. According to our results, and congruent with some of the literature demonstrating a lack of differences in virulence between MSSA and MRSA, our tertiary subexperiment comparing both UAMS-1 and USA300 showed no wound healing differences between these strains.

The data from this study suggest that both clean ischemic wounds and UAMS-1 infected ischemic wounds contribute to healing delays, though our experimental methods did not incorporate comparative testing between these two conditions. Proper comparisons, however, were tested between ischemic wounds infected with UAMS-1 and USA300, and these analyses showed comparable deficiencies in healing between the two. Though we have generally used the term “ischemia” here, based on our experience with this model [5, 6, 25–27] and the skin oxygenation data gathered from the tertiary subexperiment, the perfusion limitation created by dividing the central area is better described as “partial ischemia,” given the oxygen's prompt recovery by day 1 postinduction (Figure 2). The rapid oxygen recovery also deters us from commenting on the role, if any, that ischemia played on modulating oxygen-dependent inflammatory responses leading to pathogen death, given that, by the time we inoculated bacterial strains (POD 3), tissue oxygenation appears to have completely recovered.

Nevertheless, given statistical differences noted in the first and second substudies, it is clear that early ischemia contributes negatively to eventual wound outcomes, even when wound perfusion returns to, and persists at, normal levels for the duration of the healing process. Therefore, if ischemic conditions—such as peripheral artery disease and vasculitis—or surgical procedures—like skin flaps, stenting, and amputations—become infected, they should be promptly treated and systematically assessed for blood vessel perfusion. Also, given that no wound healing differences were seen between the two *S. aureus* strains, small cutaneous infections with either strain should be treated locally with similar approaches, with the major difference being pharmacological options obtained from antibiotic susceptibility test cultures.

Future experiments will seek to better understand how transient ischemia induced early in the wound healing process contributes to slowed wound healing overall, regardless of wound infection status. We will also assess the degree of healing deficiencies imparted by additional MSSA and MRSA strains and possibly other bacteria, in order to determine whether our findings with UAMS-1 and USA300 are generalizable. Histological immunohistochemistry will also be used to differentiate between infectious strains and the cellular immune response. Given that this study only included three histological wound parameters to differentiate healing, limitations to this study include a lack of cellular and biomarker quantification to further test differences. Other limitations include more in-depth skin oxygenation and ischemic analysis, such as transcutaneous oximetry, laser Doppler flowmetry, laser speckle contrast imaging, and thermography, along with electron microscopy of wounds for confirmation of bacterial biofilm establishment.

## Data Availability

The data that support the findings of this study are available from the corresponding author upon reasonable request.
